# Haploinsufficiency of Bcl11b suppresses the progression of ATM-deficient T cell lymphomas

**DOI:** 10.1186/s13045-015-0191-8

**Published:** 2015-07-30

**Authors:** Kerice A. Pinkney, Wenxia Jiang, Brian J. Lee, Denis G. Loredan, Chen Li, Govind Bhagat, Shan Zha

**Affiliations:** Institute for Cancer Genetics, Columbia University, 1130 St Nicholas Ave, RM 503B, New York, NY 10032 USA; Herbert Irving Comprehensive Cancer Research Center, Columbia University, 1130 St Nicholas Ave, New York, NY 10032 USA; Department of Pediatrics, Division of Hematology, Oncology and Stem Cell Transplantation, Columbia University, 1130 St Nicholas Ave, New York, NY 10032 USA; Department of Pathology and Cell Biology, College for Physicians and Surgeons, Columbia University, 1130 St Nicholas Ave, New York, NY 10032 USA; Current address: Joe DiMaggio’s Children’s Hospital, 1150 North 35th Avenue, Suite 100, Hollywood, FL 33021 USA

## Abstract

Bcl11b is a transcription factor important for T cell development and also a tumor-suppressor gene that is hemizygously inactivated in ~10 % human T cell acute lymphoblastic leukemia (T-ALL) and several murine T-ALL models, including ATM^−/−^ thymic lymphomas. Here we report that heterozygous loss of Bcl11b (Bcl11b^+/−^) unexpectedly reduced lethal thymic lymphoma in ATM^−/−^ mice by suppressing lymphoma progression, but not initiation. The suppression was associated with a T cell-mediated immune response in ATM^−/−^Bcl11b^+/−^ mice, revealing a haploid insufficient function of Bcl11b in immune modulation against lymphoma and offering an explanation for the complex relationship between Bcl11b status with T-ALL prognosis.

**Electronic supplementary material:**

The online version of this article (doi:10.1186/s13045-015-0191-8) contains supplementary material, which is available to authorized users.

## Correspondence/findings

Bcl11b is a transcription factor that is “monoallelically” inactivated in ~10 % of human T cell acute lymphoblastic leukemia (T-ALL) [1–3] and several murine T-ALL models, including ATM^−/−^ thymic lymphomas [4–6]. Both human and murine T-ALLs retain at least one intact copy of Bcl11b, indicating that Bcl11b is a bona fide haploinsufficient tumor-suppressor gene. Complete loss of the Bcl11b gene abrogates T cell development and gain of NK cell phenotype, revealing a critical role of Bcl11b in T cell lineage commitment [7–10]. Conditional inactivation of both copies of Bcl11b in double-positive (DP) T cells leads to overproduction of innate CD8^+^ single-positive (SP) T cells [11] and compromises T-regulatory cell function [12]. Yet, hemizygous loss of Bcl11b has no measurable impact on T cell function [7–10] and the mechanism by which it promotes T-ALL remains elusive.

To address this, we characterized Bcl11b^+/−^ATM^−/−^ mice [6, 13]. ATM kinase is a master regulator of the DNA damage responses [14]. ATM^−/−^ mice routinely succumb to immature T cell lymphomas sharing molecular features with human T-ALL. Of the ATM^−/−^ thymic lymphomas, 80 % hemizygously deleted Bcl11b as a result of non-reciprocal t(12;14) translocation [6, 15]. Conditional inactivation of Bcl11b in T cells via LckCre eliminates recurrent t(12;14) translocations from ATM^−/−^ thymic lymphomas, suggesting Bcl11b as the target of the large chromosome 12 deletion [16]. ATM^−/−^Bcl11b^+/−^ mice were born at expected frequency (Additional file 1: Figure S1A). Thymocyte development and peripheral T and B cell repertoire in young (4 week) ATM^−/−^Bcl11b^+/−^ mice were indistinguishable from that of ATM^−/−^ mice, displaying reduced surface TCRβ/CD3ε expression in DP cells and a partial blockade at the DP to SP transition characteristic for ATM-deficiency [6, 17] (Additional file 1: Figure S1B).

**Fig. 1:**
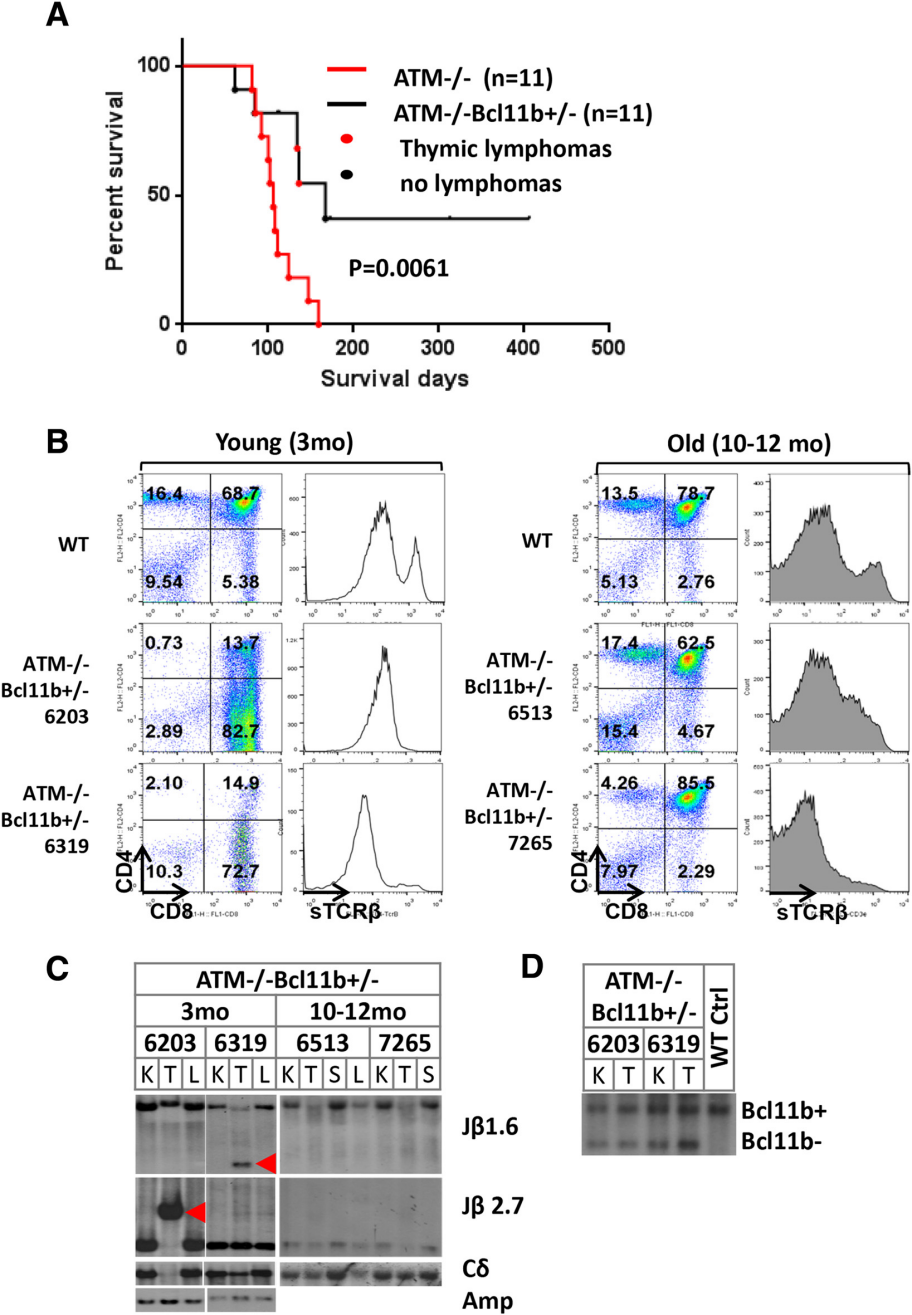
Heterozygous loss of Bcl11b suppresses the progression, but not the initiation of ATM-deficient thymic lymphomas. **a** Thymic lymphoma-free survival of ATM^−/−^ and ATM^−/−^Bcl11b^+/−^ mice. Median survival of ATM^−/−^ and ATM^−/−^Bcl11b^+/−^ cohorts was 106 and 169 days, respectively. *p* value for log-ranking test is 0.0061. **b** Representative flow cytometry analyses of the thymus from control and ATM^−/−^Bcl11b^+/−^ mice at 3 or 10 months of age. **c** Southern blot analyses of EcoRI digested genomic DNA from kidney (K), thymus (T), enlarged submandibular lymph nodes (L), or spleen (S) from ATM^−/−^Bcl11b^+/−^ mice probed with TCRβ probes (Jβ1.6 or 2.7), TCRδ constant region (Cδ) or chromosome 14 amplification region (Amp) [6]. **d** Southern blot analyses of Bcl11b locus on KpnI digested genomic DNA with Bcl11b probe [13]

While we initially expected Bcl11b-deficiency to accelerate ATM-deficient thymic lymphomas based on its frequent inactivation in T-ALLs, hemizygous-deletion of Bcl11b prevented lethal thymic lymphoma in ~50 % ATM^−/−^ mice and only 2/11 ATM^−/−^Bcl11b^+/−^ mice developed overt thymic lymphomas (Fig. 1a). The median survival of ATM^−/−^Bcl11b^+/−^ mice was significantly longer than that of ATM^−/−^ controls (167 vs. 106 days, *p* < 0.01) (Fig. 1a). Analyses of 3- and 10-month-old ATM^−/−^Bcl11b^+/−^ mice revealed clonal expansion of immature (surfaceTCRβ^low^) thymocytes in 3-month, but not 10-month-old ATM^−/−^Bcl11b^+/−^ mice (Fig. 1b, c). T cell lymphomas from 3-month-old ATM^−/−^Bcl11b^+/−^ mice retained both WT and null alleles of Bcl11b, consistent with the lack-of-LOH in T-ALL (Fig. 1d). Despite the clonal expansion in 3-month-old ATM^−/−^Bcl11b^+/−^ mice, most thymic lymphomas failed to progress to lethal disease (Additional file 1: Figure S1C), suggesting that heterozygous Bcl11b-deficiency suppresses the progression, but not the initiation of ATM^−/−^ thymic lymphomas.

**Fig. 2:**
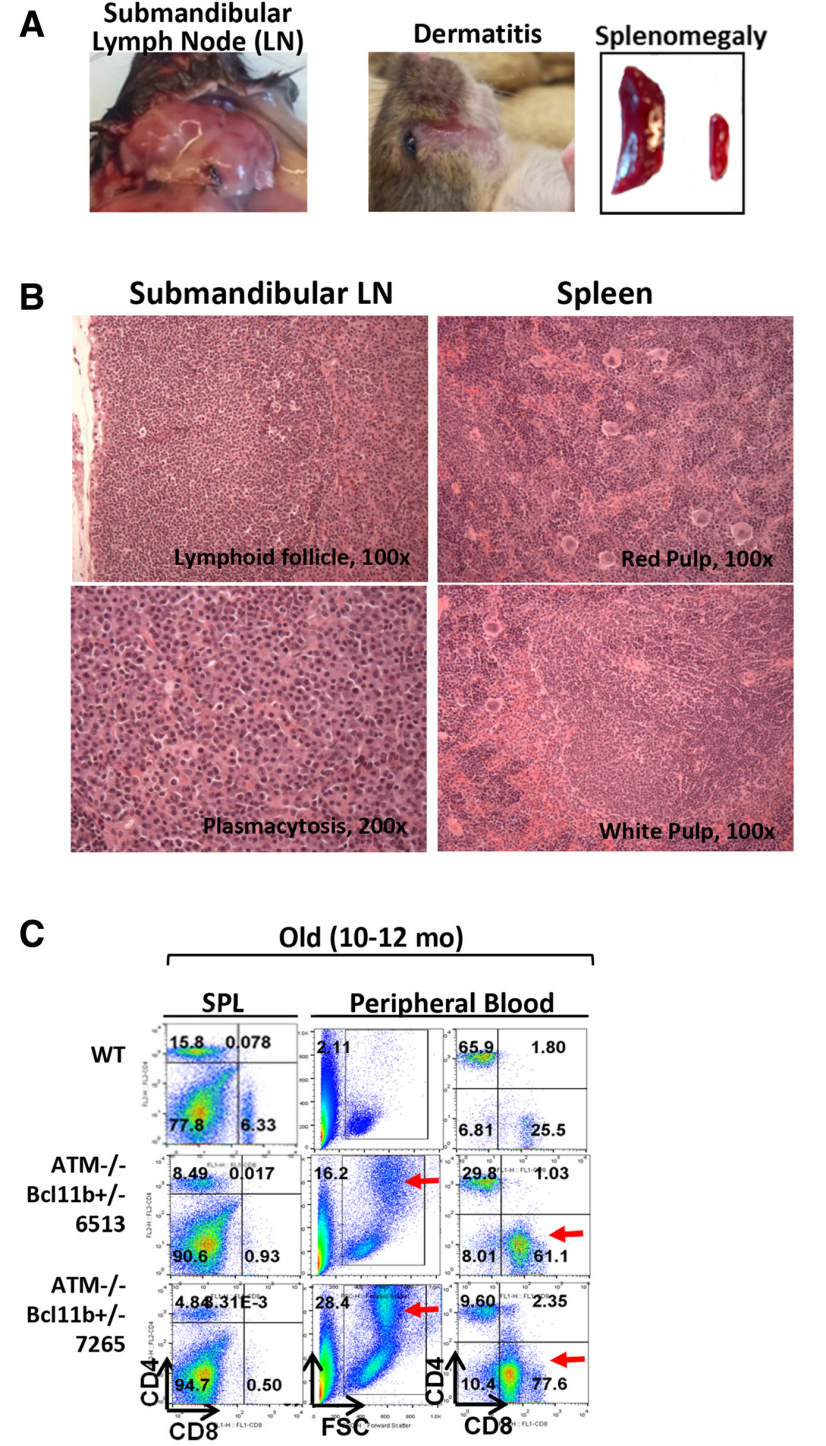
Heterozygous loss of Bcl11b induces an inflammatory/immune response in ATM^−/−^ mice. **a** Representative pictures of enlarged submandibular lymph nodes, dermatitis, and splenomegaly in ATM^−/−^Bcl11b^+/−^ mice. **b** Histopathologic analysis of the submandibular lymph nodes and spleen in ATM^−/−^Bcl11b^+/−^ mice. **c** Flow cytometry analysis shows significant enrichment of CD8^+^ SP T cells in the peripheral blood, but not the spleen of 10-month-old ATM^−/−^Bcl11b^+/−^ mice

Notably, almost all ATM^−/−^Bcl11b^+/−^ mice developed variable degrees of splenomegaly, excessive submandibular lymph node (LN) enlargement that manifested to fatal airway obstruction (non-lymphoma-related death), and dermal inflammation commencing at 2–3 months of age (Fig. 2a). In contrast to normal lymphocyte profiles in 3-month-old ATM^+/+(+/−)^Bcl11b^+/−^ mice (Additional file 1: Figure S2A and S2B), histologic analyses consistently revealed a lymphocyte-mediated inflammatory/immune disorder in ATM^−/−^Bcl11b^+/−^ mice characterized by marked plasmacytosis with reactive germinal centers (B220^+^IgM^+^ Bcl6^+^CD138^−^ B cells) in submandibular LN, reactive follicular hyperplasia of the white pulp and increased extramedullary hematopoiesis in the red pulp in the spleen (Fig. 2b and Additional file 1: Figure S2C), and acute and chronic dermal inflammation in the skin. While a cell-autonomous function of Bcl11b deletion on epidermal integrity cannot be ruled out [13], the splenic and LN changes noted raised the possibility of an autoimmune disorder, which could have contributed to the lack of tumor progression in ATM^−/−^Bcl11b^+/−^ mice. Correspondingly, 10-month tumor-free ATM^−/−^Bcl11b^+/−^ mice accumulated activated CD8^+^ T cell in PB (Fig. 2c).

Our data suggest that in an ATM-deficient background, heterozygous Bcl11b deficiency tilts immune homeostasis and limits the expansion, but not the initiation of ATM-deficient thymic lymphomas. Notably, homozygous Bcl11b deletion suppressed melanoma in murine models [18]. Given the role of Bcl11b in T cell lineage commitment, CD8^+^ T cell development, and T-reg function, our data suggest that heterozygous Bcl11b deficiency can modulate anti-tumor immune response despite the lack of measurable T cell development defects in Bcl11b^+/−^ mice [7–10]. This role of Bcl11b in immune modulation and tumor suppression might explain the discrepancies between Bcl11b status (mutation, deletion, and downregulation) and T-ALL prognosis in different studies [1–3, 19]. It also suggests that Bcl11b is likely lost later during T-ALL development, as early deletion likely causes autoimmune dysfunction, analogous to TNFAIP3 (A20) in DLBCL [20].

## Additional file

Additional file 1: Figure S1. Characterization of T cell development in ATM^−/−^Bcl11b^+/−^ mice. (A) Actual and expected offspring from breeding between Bcl11b^+/−^ATM^+/−^ and ATM^+/−^ mice. The *p* value calculated based on chi-squared test is 0.89. (B) Representative flow cytometric analysis of pre-malignant thymocytes from WT, ATM^−/−^ and ATM^−/−^Bcl11b^+/−^ mice (4–6 weeks). **Figure S2.** Analyses of autoimmunity in ATM^−/−^Bcl11b^+/−^ mice. (A) Flow cytometric analysis of the spleen, bone marrow, and lymph nodes from 3-month and 10-month-old ATM^−/−^Bcl11b^+/−^ mice. Notably, the frequency of the CD11b^+^ myeloid cells increases in the spleen of ATM^−/−^Bcl11b^+/−^ mice, consistent with increased extramedullary hematopoiesis in the red pulp seen in Fig. 2b. (B) Flow cytometric analyses of the thymus and spleen (SPL) from 3-month-old littermate ATM^+/+^Bcl11b^+/+^ (WT), Bcl11b^+/−^, ATM^+/−^Bcl11b^+/−^ mice. Double negative (DN) staining was performed on gated CD8-CD4-CD19-TCRγ/δ^−^ thymocytes. Homozygous deletion of Bcl11b dramatically increased the number of phenotypical NK cells [8, 9]. The percentage of NK1.1^+^ or CD8^+^ cells does not significantly increase in the thymus and spleen of 3-month-old Bcl11b^+/−^ mice. (C) Immunohistochemical staining with germinal center marker Bcl6 and plasma cell marker CD138 of the enlarged submandibular lymph nodes. (PDF 2082 kb)

## Abbreviations

ALL: acute lymphoblastic leukemia
ATM: ataxia telangiectasia mutated
BM: bone marrow
DN: double negative
DP: double positive
LN: lymph node
PB: peripheral blood
SP: single positive
SPL: spleen
