# Supplementary material for: Haploinsufficiency of Bcl11b suppresses the progression of ATM-deficient T cell lymphomas
Source: J Hematol Oncol. 2015 Jul 30;8:94. doi: 10.1186/s13045-015-0191-8 (PMC4518599; doi:10.1186/s13045-015-0191-8)
Supplement: Additional file 1: Figure S1. — Characterization of T cell development in ATM−/−Bcl11b+/− mice. (A) Actual and expected offspring from breeding between Bcl11b+/−ATM+/− and ATM+/− mice. The p value calculated based on chi-squared test is 0.89. (B) Representative flow cytometric analysis of pre-malignant thymocytes from WT, ATM−/− and ATM−/−Bcl11b+/− mice (4–6 weeks). Figure S2. Analyses of autoimmunity in ATM−/−Bcl11b+/− mice. (A) Flow cytometric analysis of the spleen, bone marrow, and lymph nodes from 3-month and 10-month-old ATM−/−Bcl11b+/− mice. Notably, the frequency of the CD11b+ myeloid cells increases in the spleen of ATM−/−Bcl11b+/− mice, consistent with increased extramedullary hematopoiesis in the red pulp seen in Fig. 2b. (B) Flow cytometric analyses of the thymus and spleen (SPL) from 3-month-old littermate ATM+/+Bcl11b+/+ (WT), Bcl11b+/−, ATM+/−Bcl11b+/− mice. Double negative (DN) staining was performed on gated CD8-CD4-CD19-TCRγ/δ− thymocytes. Homozygous deletion of Bcl11b dramatically increased the number of phenotypical NK cells [8, 9]. The percentage of NK1.1+ or CD8+ cells does not significantly increase in the thymus and spleen of 3-month-old Bcl11b+/− mice. (C) Immunohistochemical staining with germinal center marker Bcl6 and plasma cell marker CD138 of the enlarged submandibular lymph nodes. (PDF 2082 kb) [file 13045_2015_191_MOESM1_ESM.pdf]

SupFigure 1

A.

Genotypes		Actual	Exp(freq)
Bcl11b+/-	ATM+/+	14	1/8
	ATM+/-	33	1/4
	ATM-/-	13	1/8
Bcl11b+/+	ATM+/+	19	1/8
	ATM+/-	28	1/4
	ATM-/-	13	1/8
Total		120	

P=0.84 (χ^2Test)

B.

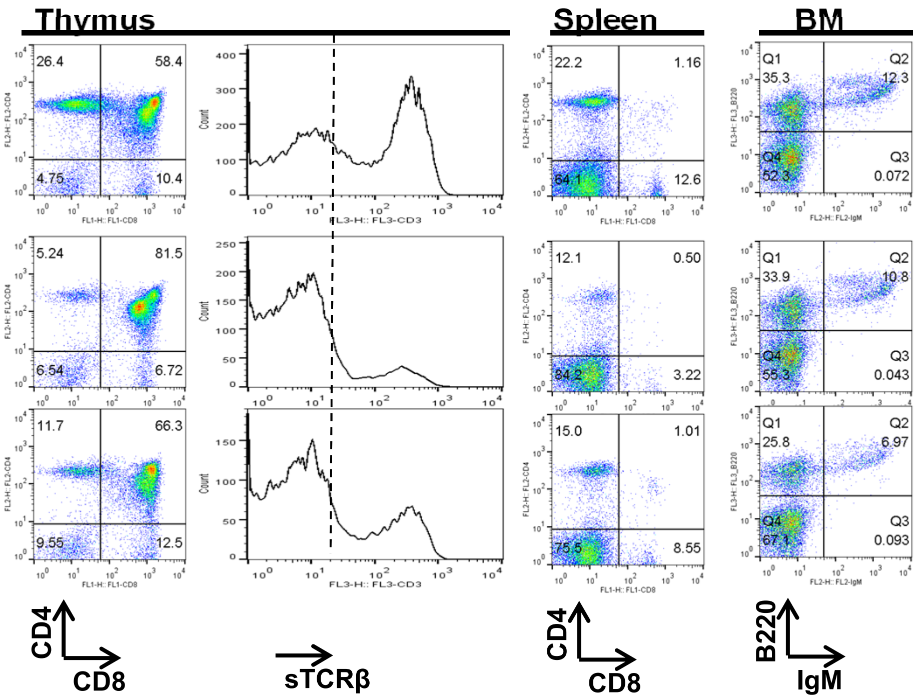

Sup. Figure 2

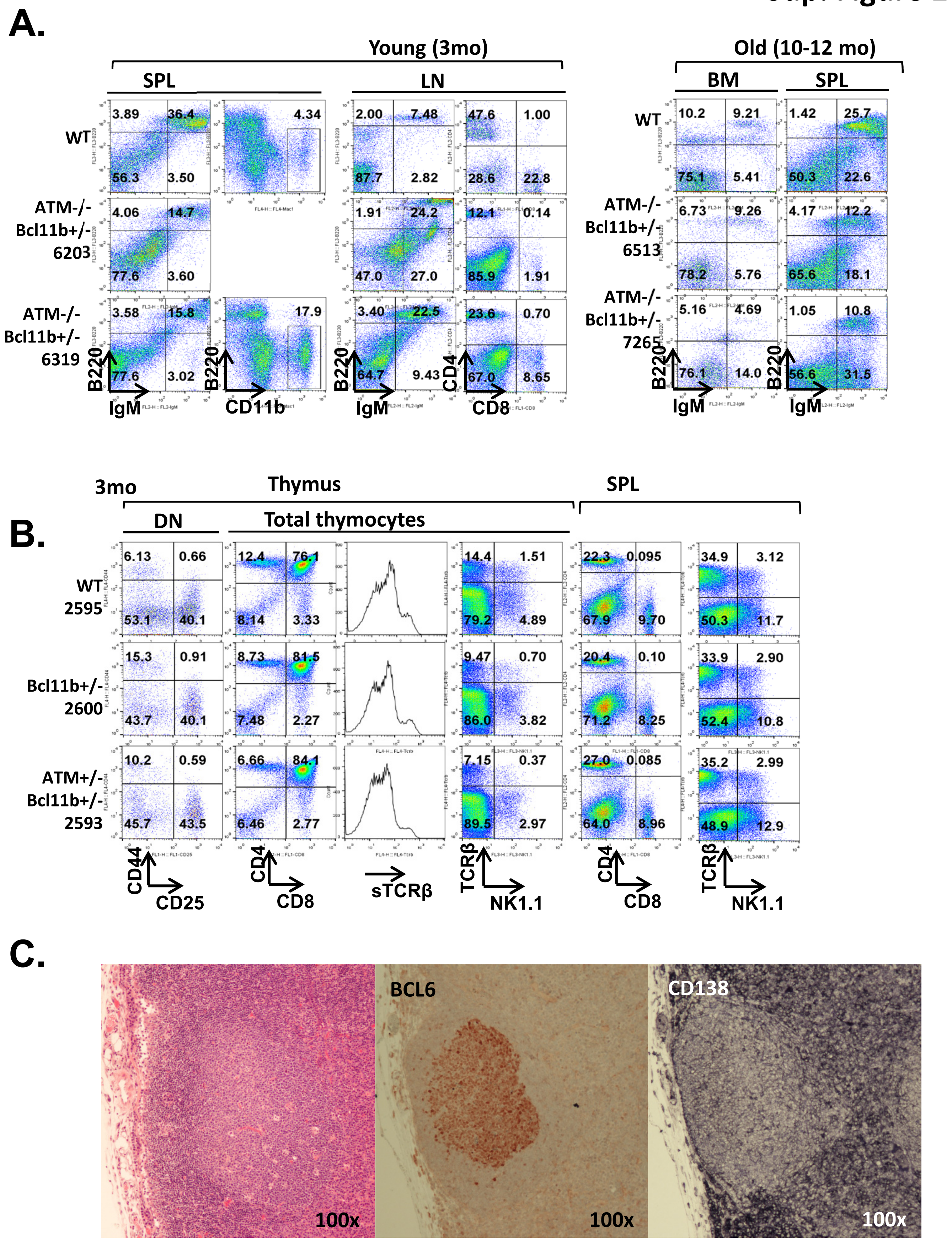
A.
Young (3mo)
Old (10-12 mo)
SPL
LN
BM
SPL
WT
ATM-/- Bcl11b+/- 6203
ATM-/- Bcl11b+/- 6319
ATM-/- Bcl11b+/- 6513
ATM-/- Bcl11b+/- 7265
3.89
36.4
56.3
3.50
4.34
2.00
7.48
87.7
2.82
47.6
1.00
28.6
22.8
10.2
9.21
75.1
5.41
1.42
25.7
50.3
22.6
4.06
14.7
77.6
3.60
1.91
24.2
47.0
27.0
12.1
0.14
85.9
1.91
6.73
9.26
78.2
5.76
4.17
12.2
65.6
18.1
3.58
15.8
77.6
3.02
17.9
3.40
22.5
64.7
9.43
23.6
0.70
67.0
8.65
5.16
4.69
76.1
14.0
1.05
10.8
56.6
31.5
B220
IgM
CD11b
CD4
CD8
B.
3mo
Thymus
SPL
DN
Total thymocytes
WT 2595
Bcl11b+/- 2600
ATM+/- Bcl11b+/- 2593
6.13
0.66
53.1
40.1
12.4
76.1
8.14
3.33
14.4
1.51
79.2
4.89
22.3
0.095
67.9
9.70
34.9
3.12
50.3
11.7
15.3
0.91
43.7
40.1
8.73
81.5
7.48
2.27
9.47
0.70
86.0
3.82
20.4
0.10
71.2
8.25
33.9
2.90
52.4
10.8
10.2
0.59
45.7
43.5
6.66
84.1
6.46
2.77
7.15
0.37
89.5
2.97
27.0
0.085
64.0
8.96
35.2
2.99
48.9
12.9
CD44
CD25
CD4
CD8
sTCRβ
TCRβ
NK1.1
C.
BCL6
CD138
100x
100x
100x

Supplementary Figure 1. Characterization of T cell development in ATM-/-Bcl11b+/- mice. (A) Actual and expected offspring from breeding between Bcl11b+/-ATM+/- and ATM+/- mice. The p-value calculated based on chi-squared test is 0.89. **(B)** Representative flow cytometric analysis of pre-malignant thymocytes from WT, $ATM^{-/-}$ and $ATM^{-/-}Bcl11b^{+/-}$ mice (4-6 week).

Supplementary Figure 2. Analyses of autoimmunity in $ATM^{-/-}Bcl11b^{+/-}$ mice. (A) Flow cytometric analysis of the spleen, bone marrow and lymph nodes from 3-month and 10-month old $ATM^{-/-}Bcl11b^{+/-}$ mice. Notably the frequency of the $CD11b^{+}$ myeloid cells increases in the spleen of $ATM^{-/-}Bcl11b^{+/-}$ mice, consistent with increased extramedullary hematopoiesis in the red pulp seen in Figure 2B. **(B)** Flow cytometric analyses of the thymus and spleen (SPL) from 3-month old littermate $ATM^{+/+}Bcl11b^{+/+}$ (WT), $Bcl11b^{+/-}$, $ATM^{+/-}Bcl11b^{+/-}$ mice. Double negative (DN) staining was performed on gated $CD8^{-}CD4^{-}CD19^{-}TCR\gamma/\delta^{-}$ thymocytes. Homozygous deletion of Bcl11b dramatically increased the number of phenotypical NK cells [8-9]. The percentage of $NK1.1^{+}$ or CD8+ cells does not significantly increases in the thymus and spleen of 3-month old $Bcl11b^{+/-}$ mice. **(C)** Immunohistochemical staining with germinal center marker Bcl6 and plasma cell marker CD138 of the enlarged submandibular lymph nodes.
